# Metal–organic framework adhesives with exceptionally high heat resistance

**DOI:** 10.1080/14686996.2024.2347193

**Published:** 2024-04-29

**Authors:** Izuru Miyazaki, Yumi Masuoka, Akitoshi Suzumura, Shinya Moribe, Hisaaki Takao, Mitsutaro Umehara

**Affiliations:** Frontier Research Office, Toyota Central R&D Labs., Inc., Nagakute, Japan

**Keywords:** Metal-organic framework, structural adhesive, heat resistance

## Abstract

We synthesized high-heat-resistant adhesives based on metal – organic frameworks owing to their high decomposition temperature and the absence of a glass transition. Heat-resistance tests were performed on adhesive joints consisting of zeolitic imidazolate framework (ZIF)-67-based adhesives and a copper substrate. The as-synthesized ZIF-67-based adhesive exhibited heat resistances at 600 and 700°C in air and nitrogen atmospheres, respectively, comparable to those of conventional high-heat-resistant polymer-based adhesives. The degradation mechanism of the ZIF-67 adhesives was investigated, and their high heat resistance was attributed to the stable existence of the ZIF-67 qtz phase in the adhesive layer at high temperatures without the formation of voids. Thus, adhesives based on ZIF-67 and other metal – organic frameworks can be applied in high-temperature industrial systems.

## IMPACT STATEMENT

By focusing on its high thermal stability and absence of glass transition, the ZIF-67 gel was found to have high potential that is comparable to existing heat-resistant adhesives.

## GRAPHICAL ABSTRACT

**Figure:**
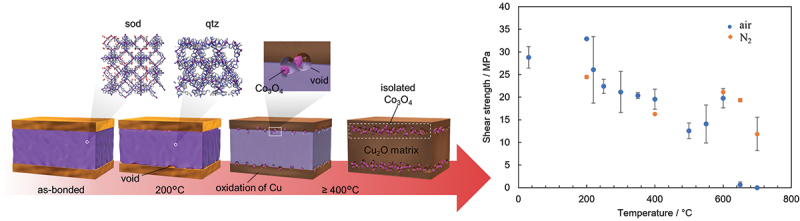

## 1. Introduction

Metal – organic frameworks (MOFs) are polymers based on coordination bonds between metal ions and organic ligands and are currently being actively investigated as potential functional materials in gas separation [1], fuel storage [2], catalysis [3], biopharmaceutical development [4], and other applications. Most previous studies of MOFs have focused on their tunable porosity [5], which is exploited in all of the aforementioned applications. Moreover, MOFs possess outstanding properties in relation to commonly used polymers. In particular, except for certain amorphous MOFs developed in recent years [6], many MOFs do not exhibit a glass transition point (softening point) [7]. The strength of conventional polymers at high temperatures is determined not by the decomposition temperature, but rather by the softening point, which is lower than this temperature [8,9]. Therefore, the absence of a glass transition indicates that MOFs may retain their original strength close to that at their decomposition temperature without softening. Furthermore, certain MOFs have decomposition temperatures much higher than the glass transition temperatures of polymers [7]. Therefore, MOF-based structures may maintain stable rigid bodies at higher temperatures in relation to polymers.

Recently, we have reported that the pressure sintering of MOFs in a gel state can realize structural adhesion [10]. Depending on the application, adhesive materials may be required to possess various properties such as electrical conductivity, thermal conductivity, and debonding ability. Thus, various functional adhesives can be realized by using MOFs with physical properties differing from those of conventional polymers. In particular, we focus on the potential of realizing heat-resistant adhesives considering the high thermal stability and absence of the glass transition in MOFs, which has not yet been investigated. Zeolitic imidazolate framework-67 (ZIF-67) is selected for verifying this concept, considering its high thermal decomposition temperature [11] and its ability to bond metals [10].

In this study, we fabricated ZIF-67-based adhesives and performed heat-resistance tests. Furthermore, we investigated the mechanism underlying the high heat resistance of the as-synthesized MOF adhesives through microstructural observations via scanning electron microscopy/energy dispersive X-ray spectroscopy (SEM/EDX), thermogravimetric/differential thermal analysis (TG/DTA), X-ray diffraction (XRD), and Fourier transform infrared (FTIR) spectroscopy.

## 2. Results and discussion

### 2.1. Heat-resistance tests

**Figure 1.**
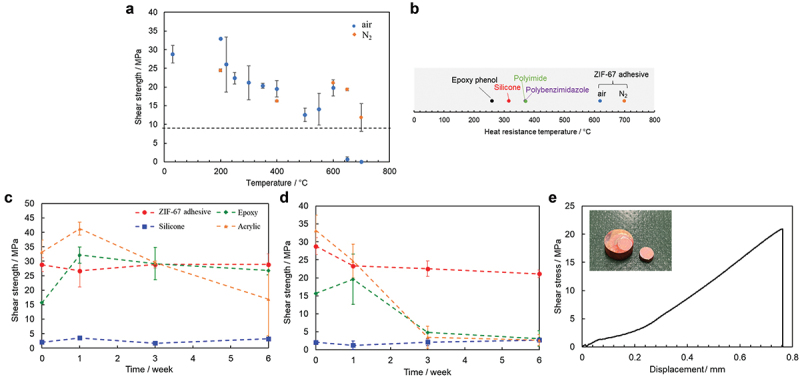
Heat-resistance of the ZIF–67 adhesive. (a) Results of the heat–resistance test conducted after 100 h. The error bars represent the standard errors. The dotted line denotes the 30% value of the initial strength. (b) Results for the as–synthesized adhesive and other polymers. Results obtained from the heat–resistance tests conducted for 6 weeks at (c) 130°C and (d) 190°C. (e) Stress–displacement curve obtained after 6 weeks from the heat–resistance test conducted at 400 °C. The inset shows a sample after testing.

Figure 1(a) shows the results of the 100-h heat-resistance test on jointed disk samples. The shear strength decreases monotonically, but slowly, with temperature; the shear strength remains above 10 MPa up to at least 600°C. According to the evaluation method of Black and Blomquist [12], ZIF-67 adhesives exhibit significantly higher heat resistances (at 600°C in air and 700°C in nitrogen) than those of conventional high-heat-resistant adhesives such as polybenzimidazole (PBI, heat resistance at 370°C [12]) and polyimide (PI, heat resistance at 370°C) [12] (Figure 1(b)). Note that inorganic adhesives generally exhibit higher heat resistance but require a more complicated curing process and longer time than organic adhesives; accordingly, their applications differ from those of organic adhesives. Therefore, inorganic adhesives were excluded here.

In general, short-term high heat resistance does not guarantee high heat resistance in the long term. For example, it has been reported that PBI exhibits excellent heat resistance when exposed for a short time (<100 h); however, it tends to decompose in hot air and undergoes rapid strength degradation upon prolonged exposure and at temperatures of ∼300°C [13]. Therefore, the long-term heat resistance of the ZIF-67 adhesive in air was investigated. The shear strength test results of the ZIF-67 adhesive and typical polymer adhesives are shown in Figure 1(c,d). At a low temperature of 130°C, almost no shear strength degradation occurs for any of the adhesives (Figure 1(c)), whereas at 190°C, the shear strength of most adhesives degrades rapidly after approximately 3 weeks (Figure 1(d)). The silicone adhesive does not undergo shear strength degradation; however, the original strength of silicone is very low. Nevertheless, the ZIF-67 adhesive maintains its shear strength of >20 MPa even after 6 weeks Figure 1(d). Even at 400°C, the shear strength test after 6 weeks shows that the adhesive joint maintains a shear strength of ∼20 MPa, indicating its surprisingly high heat resistance, unlike that observed in the case of conventional polymers (Figure 1(e)). The following sections clarify the mechanism by which the ZIF-67 adhesive develops excellent short- and long-term heat resistances.

### 2.2. Analysis of fractured surfaces

**Figure 2.**
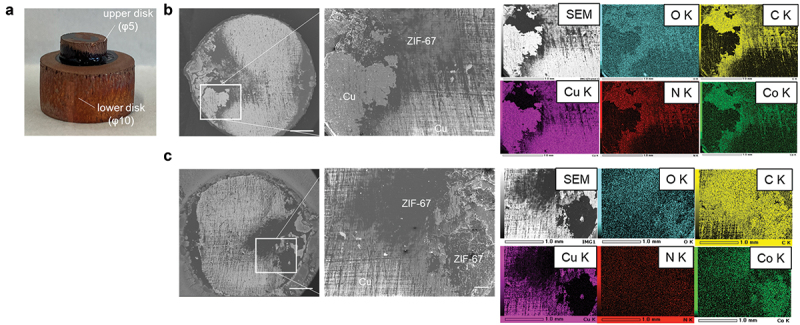
Analysis of the fractured surfaces after the heat-resistance test at 200°C in air. (a) adhesive body. SEM images (scale bar: 1 mm (original), 200 μm (magnification)) and EDX results of the (b) upper and (c) lower fractured surfaces.

To elucidate the mechanisms underlying the high heat resistance of the ZIF-67 adhesive, the fracture mode must be investigated. Figure 2 shows an SEM image of the fracture surfaces of the fabricated adhesive joint (Figure 2(a)) following the shear strength test after 100 h at 200°C in air. On the fracture surface, a phase that appears to be ZIF-67 (dark area in the SEM image) and a phase of the copper substrate (bright area in the SEM image) are observed. The EDX results for the composition of each phase are shown in Figure S1(b). The correspondence between the adhesive layer and the substrate (ZIF-67 and copper) as well as that between the adhesive layers (ZIF-67 and ZIF-67) can be observed (Figure 2(b,c)) on the upper and lower fractured surfaces, but former is primarily observed (Figure S1c). Therefore, the fracture of this sample is likely an interface fracture between the substrate and adhesive layer. It has been reported that the failure of the as-bonded ZIF-67 adhesive joint was due to cohesive failure of the internal ZIF-67 bulk layer in our prior work [10]. Therefore, the above results suggest that the failure transitioned from the internal bulk layer to the interface after heating at 200°C for 100 h.

**Figure 3.**
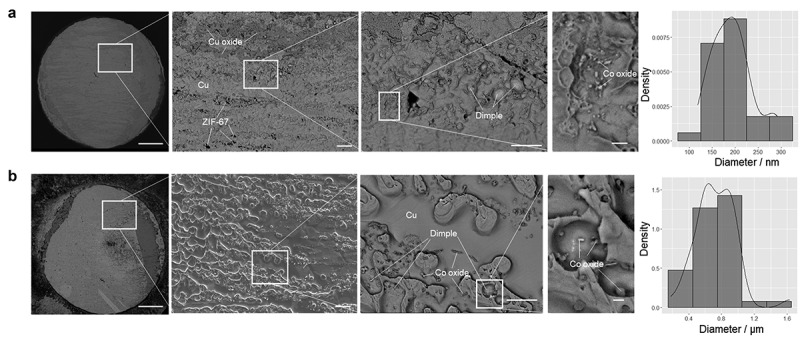
SEM images of the fractured surfaces and distribution of the diameters of the cobalt oxide particles after heat-resistance tests in air at (a) 400°C and (b) 600°C in air. The scale bars in the images represent, from left to right, 1 mm, 20 μm, 10 μm, and 1 μm. The scale bars are the same for a and b.

In joint samples subjected to a temperature of 400°C, ZIF-67 is still present in the adhesive layer, in addition to copper and copper oxide (Figure 3(a)). The EDX results for the composition of these phases are shown in Figure S2. It is difficult to observe the ZIF-67 phase on the fracture surface of the sample heated to 600°C (Figure 3(b)); however, it still seems to be present along the polishing marks on the substrate from the EDX line analysis (Figure S4). Furthermore, in the fracture surfaces of the samples subjected to temperatures above 400°C, dimple-like irregularities are observed (Figure 3(a,b)). The size of this dimple is larger at higher heating temperatures, larger at 600°C than at 400°C. From the correspondence between the dimple shapes on the upper and lower fractured surfaces (Figures S2c and S3c), one can infer that the dimple-like structures are the sheared traces of voids on the interface between the adhesive layer and copper substrate, based on the EDX results (Figures S2b and S3b). Another feature of the fracture surface after heating above 400°C is a particulate texture in addition to the dimple texture. Based on the EDX results (Figure S3b), these particles are thought to be cobalt oxide of Co_3_O_4_. The observed cobalt oxide particles are larger at higher heating temperatures, averaging 0.19 μm at 400°C, compared to 0.73 μm at 600°C (Figure 3(a,b)). As the cobalt oxides are considered to be decomposition products of ZIF-67, this result suggests that the adhesive layer deteriorates more with increasing temperature. Further identification of each phase observed at the fracture surface was conducted by subsequent XRD analysis using the ZIF-67 film as a model sample. The decomposition temperature of ZIF-67 in air has been reported to be less than 400°C [11], and this was confirmed by our TG-DTA results (Figure S5). In contrast, in the nitrogen atmosphere, no clear decomposition temperature appears, and weight loss is significantly slower than in air above 400°C (Figure S5). Therefore, the fact that ZIF-67 is still observed inside the fracture surface after heating above 400°C as shown in Figures 3(a) and S4 suggests that the decomposition of ZIF-67 progresses from the vicinity of the outer periphery of the interface in contact with air, but the progress inside the adhesive layer, which is less affected by the air atmosphere, is significantly slow on the test timescale of this study, due to which strong bonding can be retained through the adhesive layer even after prolonged heating at temperatures above the decomposition temperature of ZIF-67. From the results of the aforementioned fracture surface analysis, the formation of voids, which leads to shear failure at this location, is considered to control the high heat resistance and dependence on the atmosphere of the ZIF-67 adhesive. Therefore, the following section clarifies the process of forming such void-containing structures with joint samples using copper foil and film samples coated with the ZIF-67 adhesive as model samples (for preparation, see Experimental section).

### 2.3. Degradation behavior of adhesive joints

**Figure 4.**
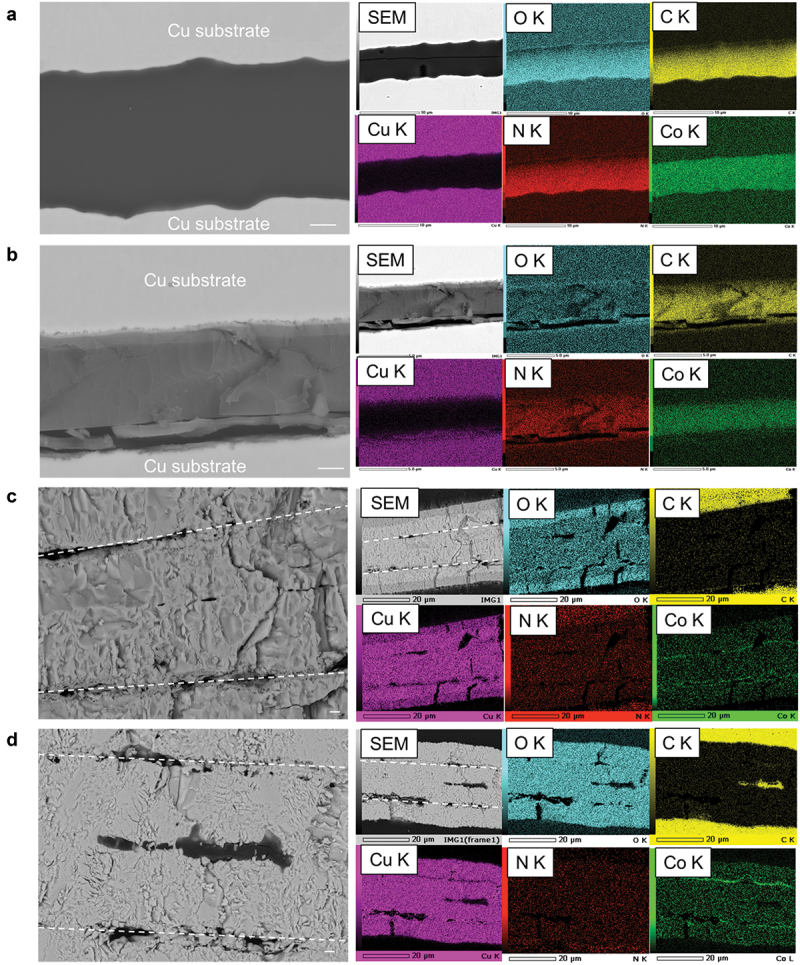
Cross-sectional SEM and EDX results. SEM images (scale bar: 1 μm) of the (a) as-bonded sample and samples subjected to the heat-resistance tests in air at (b) 200°C, (c) 400°C, and (d) 600°C. The dotted lines in (c) and (d) indicate the original adhesive–substrate interfaces.

To analyze the microstructural changes induced by the degradation of the adhesive at high temperatures, the cross-section of the jointed foil sample was observed. In the as-bonded state, the adhesive layer is smoothly attached to the uneven surface of the substrate without gaps (Figure 4(a)), whereas delamination is observed in places at the interface between the substrate and adhesive layer after heating at 200°C for 100 h in air (Figure 4(b)). This finding suggests that the degradation of the adhesive layer progresses from the interface where oxygen can easily penetrate from the outside. Although this degradation does not seem to be severe in the disk sample, as indicated in Figure 1(a), it is thought to shift the failure mode during shearing from cohesive to interfacial failure in Figure 2.

**Figure 5.**
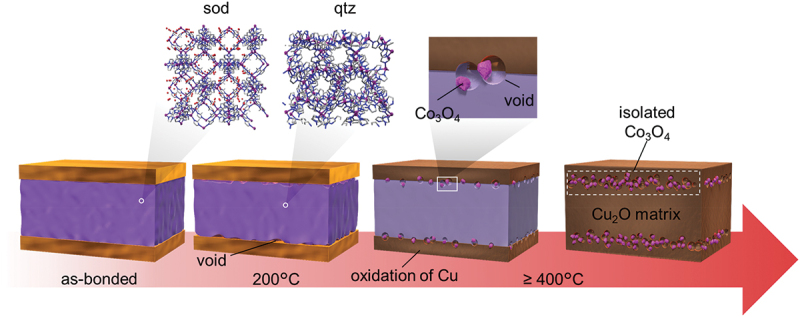
Schematic of ZIF-67 adhesive joint degradation.

When the heating temperature exceeds 400°C, the substrate and adhesive layer are assimilated and a negligible amount of cobalt or nitrogen is detected, indicating the formation of a copper oxide phase (Figures 4(c,d), S6c,d). In addition, void formation and cobalt enrichment occur at the original interface between the substrate and adhesive layer. These results correspond to the voids and cobalt oxides observed in the fracture surfaces shown in Figure 2. This cobalt enrichment was not observed in the as-bonded sample nor after heating to 200°C (Figure S6a,b). These results indicate that the adhesive layer decomposes more intensely when heated above 400°C, and the particles of cobalt oxide are generated at the adhesive – substrate interface. In addition, TG-DTA results indicate the loss of volatile components during decomposition produces voids at the adhesive – substrate interface (Figure S5b). Furthermore, the cobalt in the adhesive layer diffuses toward the adhesive – substrate interface owing to the formation of cobalt oxide. The degradation is so pronounced in the foil sample that cobalt is almost completely disappeared from inside the original adhesive layer, and cobalt oxide forms as a localized state at the original adhesive – substrate interface, as shown in Figure 4(c,d). In addition, copper in the substrate diffuses into the adhesive layer and reacts with the penetrating oxygen, resulting in oxidation and assimilation of the substrate and adhesive layer to form copper oxide. These processes are shown schematically in part of Figure 5. The diffusion pathways of cobalt and copper during heating above 400°C remain unclear. Nonetheless, one possible mechanism is as follows: in the synthesis of the ZIF-67 adhesive, 2-methylimidazole (2-MeIM) is added in excess of the stoichiometric ratio (Co:2-MeIM = 1:2), and the resulting solution is not rinsed with a solvent, leading to an excess of 2-MeIM within the ZIF-67 adhesive [10]. Given that the solidification temperature of the adhesive is lower than the boiling point of 2-MeIM, some of it is likely to remain within the ZIF-67 adhesive layer. Upon heating above the melting point of 2-MeIM, the residual 2-MeIM liquefies, providing a medium for the diffusion of the two metals, Co^2+^ and Cu^+^. However, the diffusion phenomena of metal ions in solid and liquid metal – organic structures have not yet been fully elucidated, and this hypothesis must be investigated in future studies. As mentioned in the previous section, in the disk sample, the degradation after heating above 400°C remains limited, and ZIF-67 persists inside the adhesive surface. In addition, the disk sample maintains its high shear strength after heated above 400°C, as shown in Figure 1(a). This characteristic is attributed to the thickness of the disk sample being larger (≥2 mm) than that of the foil sample (10 µm), which limits oxygen penetration to the outer edge of the adhesive layer – substrate interface. In the disk sample, as shown in Figure 3(a,b), cobalt oxide particles are observed on the fractured surface, suggesting that fracture occurs at the original adhesive – substrate interface.

### 2.4. Phase change via adhesive degradation

**Figure 6.**
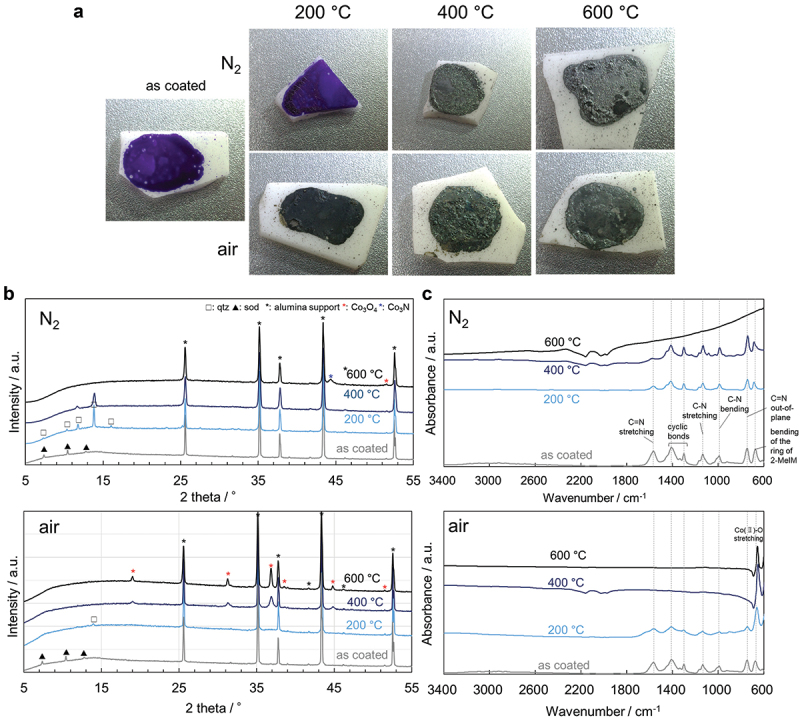
Characterization of the ZIF-67 adhesives. (a) photographs of the ZIF-67-adhesive-coated alumina substrates, (b) XRD patterns, and (c) FTIR spectra.

The ZIF-67 film sample was bright purple before heating but turned black when heated to above 200°C in air and above 400°C in nitrogen (Figure 6(a)). The XRD pattern shows a ZIF-67 sod phase after coating. After heating to 200°C in the nitrogen atmosphere, the ZIF-67 qtz phase is predominantly observed (Figure 6(b)). Upon heating to 600°C, peaks corresponding to only a small amount of Co_3_N and Co_3_O_4_ are evident. On heating in the air atmosphere, small peaks ascribed to the ZIF-67 qtz phase are observed for the sample heated to 200°C; however, Co_3_O_4_ peaks are predominantly observed for the samples heated to 400°C and above. The cobalt oxide observed in the fracture surfaces shown in Figure 3 is thought to correspond to Co_3_O_4_. The FTIR spectra indicate a ZIF-67 phase peak at 600–1600 cm^−1^ [14,15] after coating and a cobalt oxide peak at 653–655 cm^−1^ after heating to 400°C and above. These results are consistent with the XRD results. Therefore, the blackening of the film seen in Figure 6(a) is possibly due to the decomposition of ZIF-67 to black Co_3_O_4_. These processes are also shown schematically in Figure 5. The above results are consistent with the phase transition point from ZIF-67 sod to ZIF-67 qtz that reportedly occurs at ∼280°C and the thermal decomposition temperature at ∼400°C [16]. In addition, these results indicate that the ZIF-67 qtz phase is more easily pyrolyzed in air than in nitrogen, which is consistent with the TG-DTA results discussed above (Figure S5). Therefore, the adhesive layer is more stable in nitrogen than in air, preventing the formation of voids that lead to shear failure under these conditions. As a result, higher heat resistance is observed in nitrogen as shown in Figure 1(a).

## 3. Conclusion

In this study, we considered the application of high-heat-resistant adhesives synthesized using MOFs, by focusing not on their tunable pores, but rather on their high decomposition temperatures and the absence of a glass transition. We found that the synthesized ZIF-67-based MOF adhesives had significantly higher heat resistances than commonly used polymer-based high-heat-resistant adhesives. The degradation mechanism of the adhesives was found to depend on the temperature: when heated at 200°C, the ZIF-67 sod phase in the adhesive layer was converted into the ZIF-67 qtz phase. In air, part of the ZIF-67 qtz phase near the adhesive/substrate interface could decompose to Co_3_O_4_, causing delamination at the interface. When heated above 400°C, the decomposition of the adhesive layer at the adhesive – substrate interface was more intense, producing voids and cobalt oxide particles, which led to shear failure. However, a larger sample thickness could limit the extent of degradation, and the remaining ZIF-67 phase inside the adhesive layer conferred a high strength even above 400°C. Therefore, the high heat resistance of the ZIF-67 adhesive synthesized in this study was attributed to the stable existence of the ZIF-67 qtz phase in the adhesive layer up to high temperatures without the formation of voids. This result suggests that the high thermal stability of MOFs is important for high-heat-resistant adhesion. Numerous MOFs have been reported to exhibit a higher thermal stability than ZIF-67 [7], highlighting the potential of developing adhesives with even higher heat resistance. In addition, the strength of these systems must be evaluated at high temperatures to analyze the effect of the absence of the glass transition in ZIF-67. Therefore, further studies are needed to clarify the mechanical properties at high temperatures through shear strength tests and elastic modulus measurements. Furthermore, it is difficult to claim that the characteristics of adhesive bonding using ZIF-67 have been sufficiently elucidated. The chemical stability as well as mechanical properties under various stress conditions and loading rates have not been investigated. Additionally, the influences of various factors affecting these properties, such as the humidity, substrate types, and surface roughness, have not been thoroughly examined. Basic research of this nature is necessary for adhesive bonding using ZIF-67 in the future. Our findings not only open new avenues of research for MOF applications, but also promote the development of heat-resistant adhesives for industrial use.

## 4. Experimental

### 4.1. Materials

Cobalt acetate tetrahydrate (≥99.0%), 2-MeIM (≥98.0%), and methanol were purchased from Fujifilm Wako Pure Chemical Industries Ltd. (Japan) and used as received.

### 4.2. Synthesis and characterization of ZIF-67-based adhesives

The ZIF-67-based adhesives were synthesized according to the method reported by Miyazaki et al. [10]. Typically, cobalt acetate (0.6227 g) was dissolved in methanol (25 mL), to which 2-MeIM (0.821 g) was added, followed by stirring overnight at room temperature. The resulting colloidal solution was dried in air at room temperature for 48 h. As a result, a non-flowing clay-like gel was obtained. This gel contained ZIF-67 amorphous nanoparticles with a radius of ∼40 nm that were loosely aggregated because of weak van der Waals forces [17]; these particles could be crystallized through heat treatment [18]. The gel was subjected to thermogravimetric/differential thermal analysis (TG/DTA) to investigate the thermal stability of this system; the analysis was performed on an alumina pan using a Rigaku D-DSC8230/TG8120IRH system at a temperature increase rate of 5°C/min from room temperature to 700°C in an air or nitrogen atmosphere.

### 4.3. Adhesion

Copper was chosen as the adherend material for this study. This is because the ZIF-67 adhesive is highly selective for the adherend material, and only copper-to-copper bonding has been reported so far [10]. Oxygen-free copper disks (Ra = 0.8) of dimensions φ10 × 5 mm (lower disk) and φ5 × 2 mm (upper disk) were used as adherends for the strength tests, and an oxygen-free copper foil of 10 μm thickness was utilized for microstructure observations. Furthermore, a foil-shaped sample was employed to analyze the microstructural changes due to thermal degradation during the preparation of the sample for SEM. The adherend was subjected to ultrasonic cleaning with acetone for 10 min, pickling with 7% hydrochloric acid for 30 s, and dehydration cleaning with acetone for 3 min before being used for bonding. Bonding was performed using a small hot-pressing machine (G-12RS-500, Takachiho Seiki) equipped with a heating device on a die. Approximately 5 mg of the synthesized gel was applied to one copper adherend on the bottom die and the other copper adherend on the top. The bonding temperature was 250°C, the applied pressure was 45 MPa, and the bonding time was 300 s. The ZIF-67 adhesive used in this study has a large volume shrinkage rate, and so far we have not been able to control the thickness to a predetermined value. Instead, by observing the cross section of the produced adhesive, we have confirmed that the thickness will be about 2 µm to 5 µm under the present bonding conditions.

### 4.4. Heat-resistance tests and characterization of adhesive joints

For the heat-resistance tests, the jointed disk samples were heated in an electric furnace from 200 to 700°C in an air or nitrogen atmosphere for a specified time (100 h or 6 weeks). The samples were then subjected to a shear strength test in an air atmosphere at room temperature. The shear strength test was performed at a stroke rate of 1 mm/min using a self-made measurement system with an IMADA force gauge and a motorized stand. We evaluated the heat resistance of the samples according to the method reported by Blomquist et al. [12]. In their method, the heat resistance of several adhesives was evaluated by varying the temperature, and the temperature at which the adhesive reached 30% of its initial shear strength after degradation for 100 h was chosen.

To clarify the phase change of the adhesive layer during degradation while avoiding the effects of destructive testing, the ZIF-67 films were prepared as follows. A colloidal solution of 0.5 mL of ZIF-67 was dropped onto an alumina substrate heated to 150°C on a hot plate and heated for 3 min to form a ZIF-67 film. The resulting films were then heated under the same conditions applied for the heat-resistance test. XRD and FTIR experiments were performed by using these coated samples. XRD patterns were recorded using a Rigaku MiniFlex system with CuKα radiation and a step size of 0.02° at a scan speed of 10°/min. FTIR spectroscopy was performed using a Thermo Scientific total reflection FTIR spectrometer (Nicolet iS50). The XRD and FTIR analyses were performed with the ZIF-67 adhesive coated on an alumina substrate, rather than on the fractured surface of the ZIF-67 adhesive, owing to the phase changes and changes in surface smoothness induced during the shear strength test. SEM images were recorded using a JEOL JSM-7000F system at an acceleration voltage of 10 kV, and EDX analysis was performed at 15 kV. For cross-sectional observations, the as-bonded and 200°C- and 400°C-heated foil samples were sandwiched between an additional copper foil (0.2 mm thick) to secure the foil samples. The 600°C-heated foil samples were directly embedded in an epoxy resin. Each sample was sectioned at room temperature using an ultramicrotome (Leica Microsystems EM UC7i-FC).

## Supplementary Material

Supplemental Material

## Data Availability

The data generated and/or analyzed during the current study are available from the corresponding author on reasonable request.
