# Supplementary material for: Metal–organic framework adhesives with exceptionally high heat resistance
Source: Sci Technol Adv Mater. 2024 Apr 29;25(1):2347193. doi: 10.1080/14686996.2024.2347193 (PMC11149564; doi:10.1080/14686996.2024.2347193)
Supplement: Supplemental Material [file TSTA_A_2347193_SM5118.docx]

Supporting Information

Metal–organic framework adhesives with exceptionally high heat resistance

Izuru Miyazaki*, Yumi Masuoka, Akitoshi Suzumura, Shinya Moribe, Hisaaki Takao, Mitsutaro Umehara

Corresponding author: miyazaki-izuru@mosk.tytlabs.co.jp


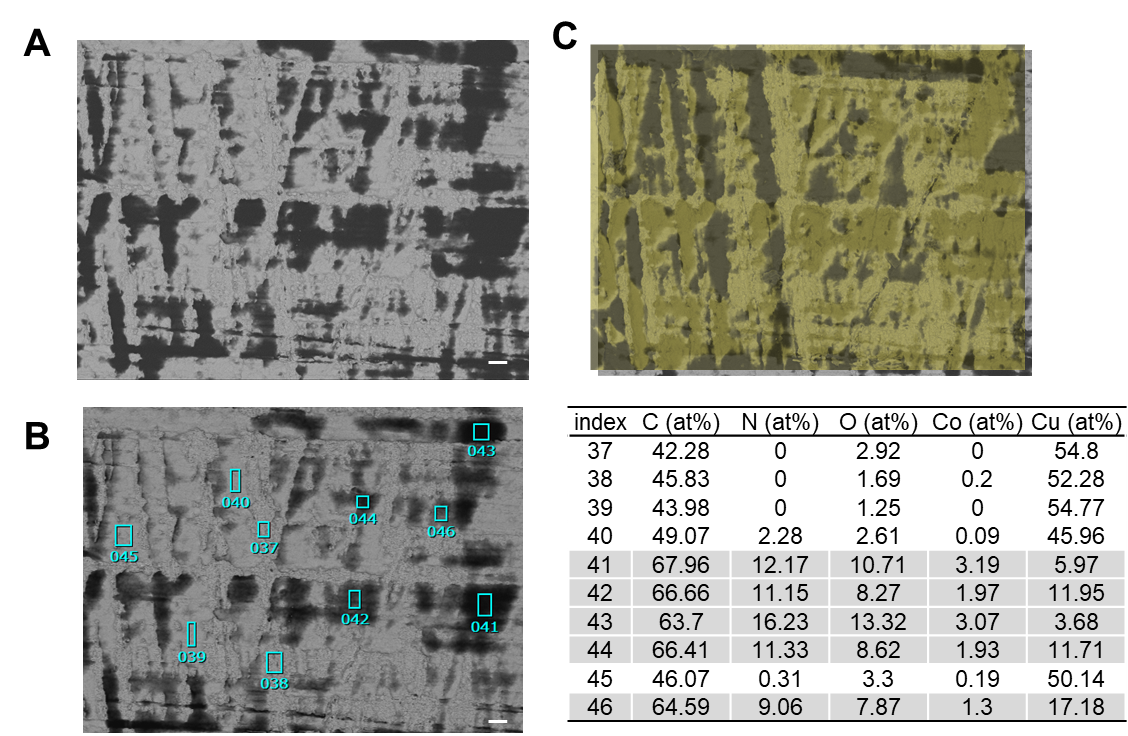
Fig. S1. SEM and EDX results for the fractured surfaces after the heat-resistance test at 200 °C in air. A) SEM image (scale bar: 10 μm), B) EDX profiles (scale bar: 10 μm), and C) superimposed images of the upper and lower fractured surfaces.


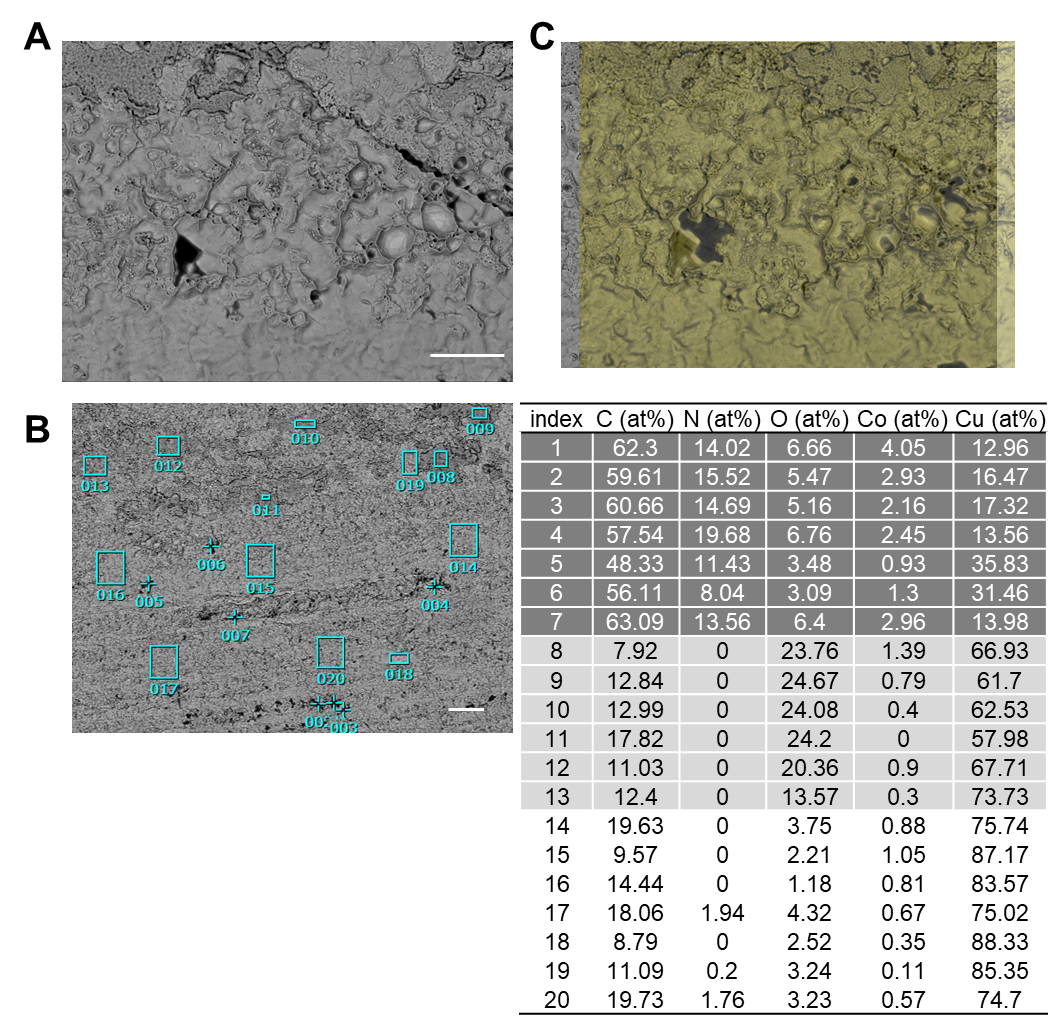
**Fig. S2. SEM and EDX results for the fractured surfaces after the heat-resistance test at 400 °C in air. A)** SEM image (scale bar: 10 μm), **B)** EDX profiles (scale bar: 20 μm), and **C)** superimposed images of the upper and lower fractured surfaces.


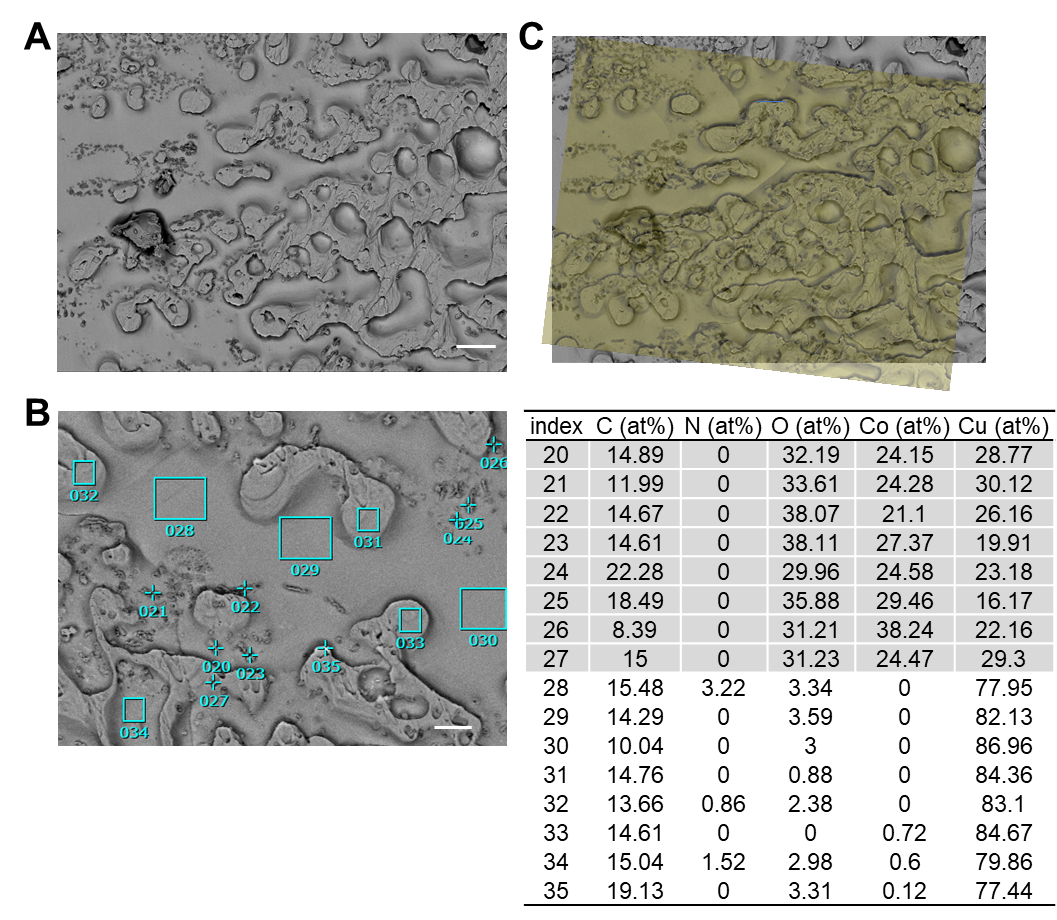
**Fig. S3. SEM and EDX results for the fractured surfaces after the heat-resistance test at 600 °C in air. A)** SEM image (scale bar: 10 μm), **B)** EDX profiles (scale bar: 20 μm), and **C)** superimposed images of the upper and lower fractured surfaces.

**
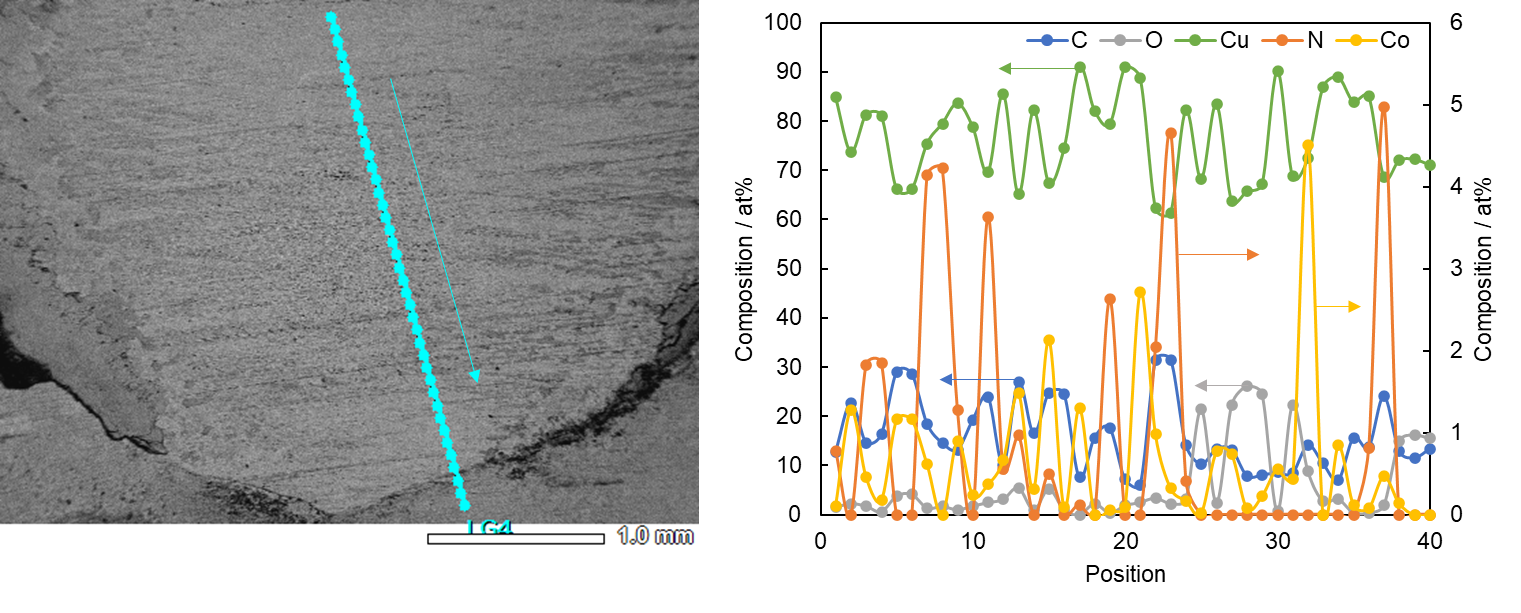
Fig. S4. EDX line analysis of the fractured surfaces after the heat-resistance test at 600 °C in air.**


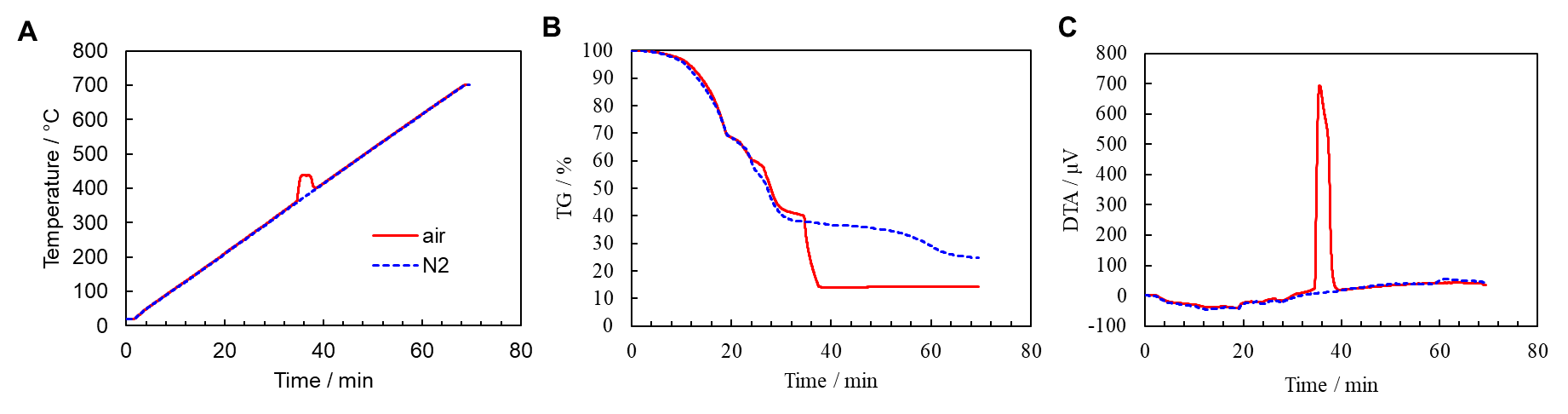


Fig. S5. Thermogravimetric results of the ZIF-67 adhesive. A) Temperature, B) TG, and C) DTA profiles.


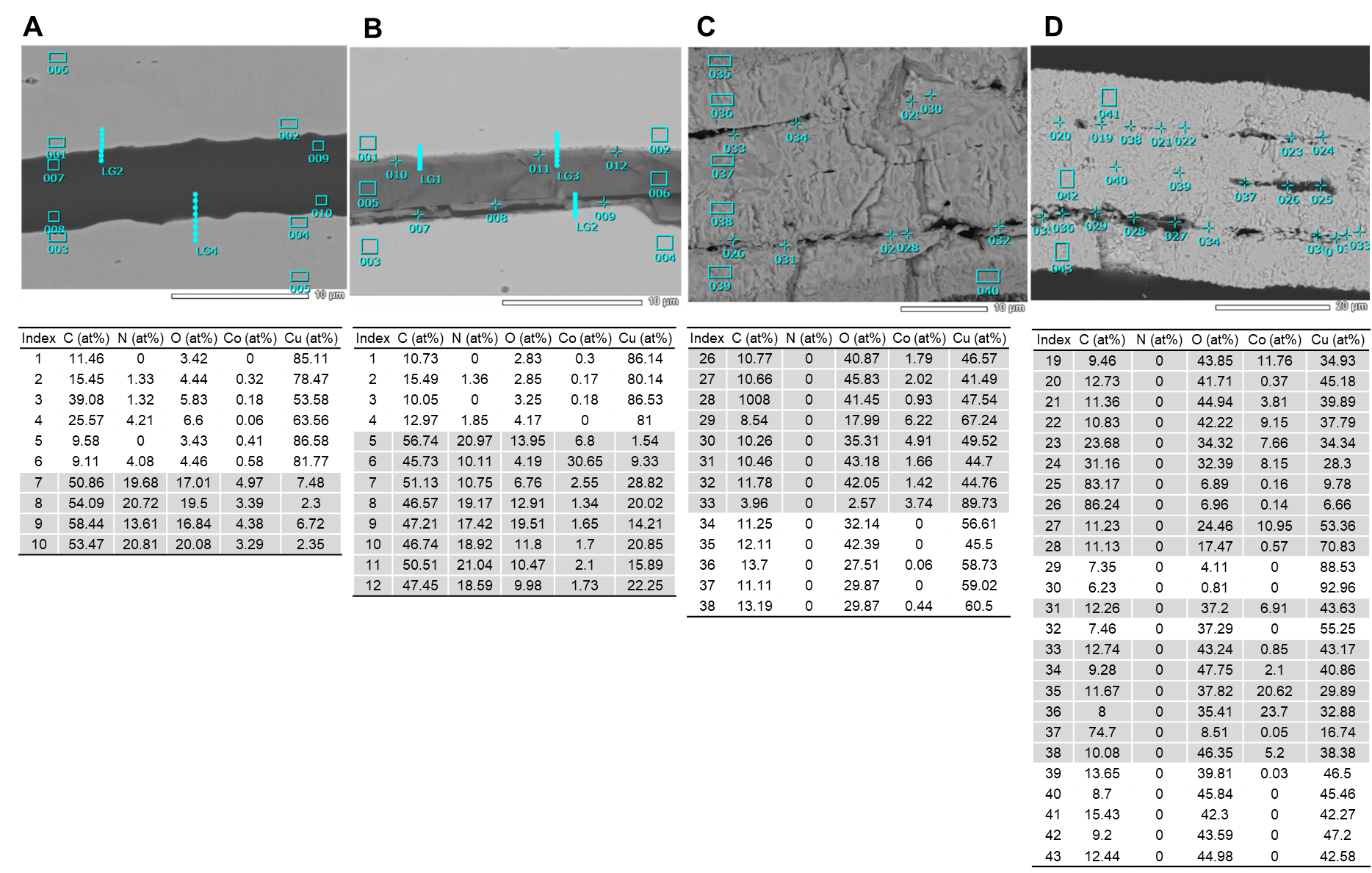


Fig. S6. Cross-sectional SEM and EDX results. SEM images of the A) as-bonded sample and samples subjected to the heat-resistance tests at B) 200 °C, C) 400 °C, and D) 600 °C.
